# Circadian function in patients with advanced non-small-cell lung cancer

**DOI:** 10.1038/sj.bjc.6602859

**Published:** 2005-11-01

**Authors:** R D Levin, M A Daehler, J F Grutsch, J Quiton, C G Lis, C Peterson, D Gupta, K Watson, D Layer, S Huff-Adams, B Desai, P Sharma, M Wallam, M Delioukina, P Ball, M Bryant, M Ashford, D Copeland, M Ohmori, P A Wood, W J M Hrushesky

**Affiliations:** 1 Cancer Treatment Centers of America® (CTCA) at Midwestern Regional Medical Center, Zion, IL, USA; 2 WJB Dorn Veterans Affairs Medical Center, Columbia, SC, USA

**Keywords:** circadian function, non-small-cell lung cancer, rest/activity function, sleep quality, quality of life, actigraphy

## Abstract

This study aimed to evaluate whether patients with advanced non-small-cell lung cancer experience disrupted rest–activity daily rhythms, poor sleep quality, weakness, and maintain attributes that are linked to circadian function such as fatigue. This report describes the rest–activity patterns of 33 non-small-cell lung cancer patients who participated in a randomised clinical trial evaluating the benefits of melatonin. Data are reported on circadian function, health-related quality of life (QoL), subjective sleep quality, and anxiety/depression levels prior to randomisation and treatment. Actigraphy data, an objective measure of circadian function, demonstrated that patients' rest–activity circadian function differs significantly from control subjects. Our patients reported poor sleep quality and high levels of fatigue. Ferrans and Powers QoL Index instrument found a high level of dissatisfaction with health-related QoL. Data from the European Organization for Research and Treatment for Cancer reported poor capacity to fulfil the activities of daily living. Patients studied in the hospital during or near chemotherapy had significantly more abnormal circadian function than those studied in the ambulatory setting. Our data indicate that measurement of circadian sleep/activity dynamics should be accomplished in the outpatient/home setting for a minimum of 4–7 circadian cycles to assure that they are most representative of the patients' true condition. We conclude that the daily sleep/activity patterns of patients with advanced lung cancer are disturbed. These are accompanied by marked disruption of QoL and function. These data argue for investigating how much of this poor functioning and QoL are actually caused by this circadian disruption, and, whether behavioural, light-based, and or pharmacologic strategies to correct the circadian/sleep activity patterns can improve function and QoL.

Every cell in our body has oscillators involved in the sending and receiving of signals that regulate the physiological economy of the body. Many of these oscillators have a circadian period, and their synchronisation integrates the many physiological and behavioural processes required to meet the challenges of daily life (Mormont and Levi, 2003).

The experimental destruction of an animal's rest–activity rhythms accelerates tumour growth, while restoring normal circadian function enhances the survival benefits of chemotherapy (Filipski *et al*, 2003). However, there has been surprisingly little data collected on the circadian function of cancer patients. What little is known confirms the preclinical data that links distorted circadian function with accelerated tumour progression. Hrushesky *et al* (1985) first documented abnormal circadian patterns among cancer patients. Mormont *et al* (2000) found that a flattened rest–activity circadian rhythm in metastatic colon cancer patients was an independent predictor for treatment failure and premature death. Similarly, an abnormally flat circadian cortisol rhythm in metastatic breast cancer patients was an independent predictor of survival (Sephton *et al*, 2000).

Although direct measurements of circadian function in cancer patients are sparse, data on behaviours or symptoms linked to disrupted circadian function, such as sleep quality and fatigue, is relatively extensive. For example, the subjective sleep quality of cancer patients approaches levels found in insomniacs (Silberfarb *et al*, 1993; Mormont and Waterhouse, 2002; Sephton and Spiegel, 2003). Consequently, disturbances in sleep patterns may account for many of the symptoms that plague cancer patients – fatigue and decreased immune function – as well as emotional problems such as irritability, depression and decreased pleasure in work and social activities (Sheely, 1996).

Fatigue is linked to disrupted sleep rhythms and the prevalence of fatigue rises to over 80% in patients undergoing chemotherapy (Lee *et al*, 2004). Indeed, approximately 50–75% of cancer patients have fatigue levels that impair health-related quality of life (QoL) at diagnosis. Consequently, it is not surprising that treatment-related fatigue is not necessarily ameliorated by erythropoietin agents and aerobic exercise.

The natural history of neoplastic diseases reveals a link between a disrupted circadian/sleep functions and many of the symptoms that degrade the patients' QoL. It is uncertain whether cancer treatment affects circadian function (Lee *et al*, 2004). There is emerging evidence that suggests cancer patients are at high risk for problems associated with disrupted circadian function; poor sleep quality, fatigue, and decreased QoL.

During the past several years, a series of treatment innovations have promised to significantly prolong the survival of cancer patients. For example, the introduction of platinum compounds into the treatment of advanced non-small-cell lung cancer (NSCLC) has significantly prolonged survival of patients who are either refractory to, or relapse following first-line chemotherapy (Evans, 2004). None of these protocols, however, have exploited the circadian rhythms that regulate the timing of gene activity and cell function. Today, investigators have discovered that the rest/activity cycle is a reliable marker of the circadian system (Mormont and Levi, 2003). There is accumulating preclinical and clinical evidence that patients with advanced cancer placed on chronotherapeutic protocols have significantly longer lifespan, higher QoL scores and experience fewer and less-intense symptoms (Mormont *et al*, 2000).

The chronobiotic, clock-resetting agent and sleep-facilitating hormone melatonin plays a significant role in the synchronisation of the sleep/wake cycle. Distortions in the production or release of melatonin have been linked to a variety of symptoms such as insomnia and daytime drowsiness (Haimov and Lavie, 1997). Others have shown that diminished circadian amplitudes and dampened night time melatonin release characterise the melatonin circadian dynamics of patients with cancer (Lewy *et al*, 1980; Danforth *et al*, 1985; Bartsch *et al*, 1992). Lewy has repeatedly demonstrated the capacity of orally administered melatonin to reset the circadian clock (Lewy *et al*, 1999). The medical literature reports that melatonin produces a variety of favourable outcomes in oncology patients. Lissoni's group reports that melatonin reduces the toxicity of various chemotherapeutic agents, including Cisplatin, Etoposide, Anthracyclines, and 5-Flourouracil. They found a statistically significant reduction in treatment-related adverse events, such as myelosuppression, neurotoxicity, nephrotoxicity, cardiotoxicity, and asthenia (Lissoni *et al*, 1992, 1994, 1997, 1999).

We have designed a multicentre trial to evaluate the chronotherapeutic role of melatonin in the treatment of stage III and IV NSCLC and to analyse the relationships between circadian function, fatigue, insomnia and overall QoL. This interim analysis evaluated the circadian function, sleep quality, and levels of fatigue prior to treatment of the first 33 study patients. These data are the initial step in evaluating the chronotherapeutic role of melatonin in patients with advanced lung cancer.

## PATIENTS AND METHODS

### Patients

The study was conducted at Cancer Treatment Centers of America at Midwestern Regional Medical Center (MRMC) and WJB Dorn Veterans Affairs Medical Center (VAMC). Patients, between the ages of 18 and 80 years, who had a pathologically confirmed diagnosis of Stage IIIA, IIIB–IV NSCLC, bidimensionally measurable disease and an Eastern Cooperative Oncology Group (ECOG) performance status of 0, 1, and 2 were included. Patients who had failed chemotherapy were acceptable. Ineligible patients had medical conditions that precluded the administration of chemotherapeutic agents such as inadequate renal function with serum creatinine >221 *μ*mol l^−1^, inadequate hepatic function with bilirubin >34.2 *μ*mol l^−1^, severe uncontrolled heart failure, hypertension, arrhythmia, angina, lung disease, meningeal carcinomatsis, uncontrolled infection, and uncontrolled brain metastases. The patients signed an Informed Consent indicating that they were aware of the investigational nature of the study and the randomised study design. Institutional Review Boards at MRMC and VAMC approved this study.

### Procedures and summary of measurement tools

#### Actigraphy measurements of rest–activity cycles

A watch-like wrist actigraph (Ambulatory Monitoring, Inc., AMI), worn on the nondominant wrist, was used to record a patient's movement pattern over a 4–7-day period (Hauri and Wisbey, 1992; Hilliker *et al*, 1992). Patients at VAMC had all activity measurements performed in the week prior to therapy in the ambulatory/home setting. Owing to the tertiary referral nature of the MRMC base, actigraphy was performed immediately before or during and immediately following the first three chemotherapy cycles. Midwestern Regional Medical Center patients were inpatients during this measurement span.

#### The Pittsburgh Sleep Quality Index (PSQI)

The PSQI is a self-reported questionnaire that measures sleep quality and quantity over a 1-month period. The PSQI contains 19 items that comprise an overall sleep score, and it produces separate component scores in seven areas: subjective sleep quality, sleep latency, sleep duration, habitual sleep efficiency, sleep disturbances, use of sleeping medication, and daytime dysfunction. The component scores are combined to produce the *Global Sleep Quality Score* ranging from 0 to 27.

#### The European Organization for Research and Treatment of Cancer Quality of Life Core Questionnaire (EORTC QLQ-C30)

The EORTC QLQ-C30 was developed to measure a patient's capacity to fulfil the activities of daily life at the workplace and at home. Five function scales measure physical, role, emotional, social, and cognitive function, and three symptom scales measure fatigue, nausea, and pain. There is one global health/overall QoL scale and six single questions on symptoms and financial difficulties. All scores range from 0 to 100. High scores on overall QoL and the five functioning scales imply normal or unlimited function, whereas high scores on the symptom scales imply higher, more intense symptoms.

To interpret the clinical significance of the scores, we characterised differences from the normal population as large, moderate, or small according to data from Osoba *et al*, who obtained data from patient interviews. This research reported that a small change for the better or worse followed a shift of 5–10 points, while a 20-point increment or decrement in score reflected a large change in patients' QoL (Osoba *et al*, 1998).

#### Ferrans and Powers QoL Index (QLI)

The QLI is a self-reported questionnaire that measures QoL based on a patient's level of satisfaction with respect to various aspects of life, and the importance of certain aspects in his/her life. We used Cancer Version III, which asks the patient to rate satisfaction with such aspects of life as health, energy, personal relationships, and physical functioning. This version also asks the patient to rate the importance of aspects such as health, pain, independence, and personal relationships. The scores range from 0 to 30, with higher scores indicating greater satisfaction with life.

### Statistical analysis

Descriptive statistics such as mean and standard error were computed for numeric demographic factors, Sleep Index, and QoL scores. Either analysis of variance or the nonparametric counterpart, Kruskall–Wallis test, whichever was appropriate was used to compare scores differences among groups. These analyses were implemented in SAS v.2 (Cary, NC, USA).

A detailed description of the actigraphy measurement has been included in Appendix I. Owing to the differences in the setting of actigraphy monitoring between centres, outpatient/home, prior to chemotherapy in VAMC *vs* in-patient and during chemotherapy for MRMC/Zion, all analyses were performed such that centre differences were not ignored.

## RESULTS

### Patient characteristics

There were systematic differences in demographic and clinical variables in study participants by site. The MRMC site patients were younger than VAMC (56.71 *vs* 71.75, years, respectively; *t*=4.39 *P*<0.01) and higher proportion of the MRMC patients had prior chemotherapy than VAMC (14 of 21 *vs* four of 12, respectively; *χ*^2^=3.42, *P*=0.06). Of the 21 MRMC patients, 12 were female patients, while VAMC only treated males (*χ*^2^=10.77, *P*<0.01). A significantly higher proportion of MRMC patients had stage IV (80%) disease than VAMC (20%) (*χ*^2^=5.87, *P*=0.02). Finally, MRMC collected actigraphy data during chemotherapy in hospitalised patients, while VAMC data were collected at the patient's home prior to chemotherapy.

### Sleep and activity patterns

#### Actigraphy data

Table 1 summarises the actigraphy data during putative daily wake spans and compares 33 cancer patients with a reference population of 35 normal adults. In the absence of available actigraphy database of gender and age-matched individuals, we considered the actigraphy database generated by Ambulatory Monitoring Inc., 2002 as our reference population. These data were obtained from a heterogenous group of 35 adults, who were 20–50 years old, who worn actigraphs for 3 days on the average and had no known disease or chronic condition. We found that in all parameters, cancer patients were significantly different than the reference population. Table 2 depicts actigraphy data during putative daily sleep spans. As during the wake span, the cancer patients differed from the reference population in all parameters. In both wake and sleep cycles and for all parameters, cancer patients actigraphy data indicate fractured and disrupted circadian function.

#### Differences in actigraphy baseline data by site

There were significant differences in actigraphy results by site. Actigraphy data were recorded and obtained in the in-patient setting at MRMC, while obtained in the patients' home for VAMC patients. Consequently, a simultaneous comparison of circadian parameters (Bingham test) found that the average circadian rhythms of patients at both sites were significantly different (*F*=16.86, *P*<0.01). For example, VAMC patients had higher circadian amplitudes, indicating more robust rhythms (*F*=4.46, *P*=0.04). WJB Dorn Veterans Affairs Medical Center patients were more active as indicated by their higher peak activity (*F*=4.84, *P*=0.036). Also, the day–night activity difference was greater in VAMC patients as indicated by the higher I<O (90.0±2.2 vs 78.9±2.4). They also had more stable circadian rhythm pattern as exhibited by the higher mean 24-h autocorrelations (*F*=5.41, *P*=0.029).

#### Pittsburgh Sleep Quality Index

Data from each PSQI showed that the lung cancer patients scored consistently higher than a control group in all seven-component scores. Consequently, the mean *Global Sleep Quality Score* for the study population is 10.6, which is more than 2.0 points from the >8 score and much higher than the score of 5 that identifies poor sleep quality in cancer-free individuals (Carpenter and Andrykowski, 1998) (Table 3).

While there was no statistically significant difference by site for the Global Sleep Quality, there were statistically significant differences in two of the seven components of the PSQI. Sleep quality was statistically significantly better among veteran patients at VAMC (1.5±0.20) than MRMC patients (2.3±0.3; *F*=5.60, *P*=0.025). Moreover, MRMC patients used more sleep medications than VAMC (*F*=5.88, *P*=0.022).

### Quality of life

Patients with advanced NSCLC reported lower scores for all EORTC-QLQ-C30 domains when compared to population-based controls (Table 4).

Despite the differences in the sites by patients' age and clinical history, there were few differences in the EORTC instrument scores. Nonetheless, VAMC showed a statistically significant lower physical function, 47.3 compared to MRMC's 69.5 (*P*<0.001), which may reflect that VAMC patients were older. In addition, there was a 22-point difference in the Dyspnoea item, 38.1 for MRMC compared to 60.6 for VAMC (*P*<0.014). This indicates that VAMC patients experienced worse dyspnoea condition as compared to MRMC patients.

Table 5 reports the results from the Ferrans and Powers QLI, and compares the mean scores of the study population with population-based data provided by Ferrans. MRMC patients' score of 12.5 was statistically significantly lower than the VAMC score of 18.7 (*P*=0.04). Interestingly, MRMC patients' capability of completing the physical activities of daily life was statistically significantly higher in the EORTC physical domain, but their dissatisfaction with their capacity to fulfil the activities of daily living was much higher than the VAMC patients.

### Circadian function and QoL outcomes by performance status

In NSCLC, patients with a PS 2 have a much poorer prognosis and experience lower rates of tumour response to therapy than patients with PS of 0 and 1 (Hoang *et al*, 2005).

Several circadian sleep/wake parameters were computed in order to assess and compare sleep characteristics of patients with different Performance Status. The mean duration of long sleep episodes fell from 129 min for patients with PS 0–96.5 min in PS 2 patients (*P*<0.05). This indicates that patients with poor performance status (PS 2) tend to have more disturbed sleep during the night. These long sleep spans, however, are short compared to those of normal adults, which average 225 min. Similarly, the mean duration of activity during wakefulness was unaffected by PS score; however, patients with PS 2 tend to be less active than patients with PS 1. In the normal population, the duration of mean activity during wakefulness was 182.6 min, which is nearly 60 min longer than our PS 0 patients. Although the number of sleep interruptions nightly was not statistically significant among PS levels, there were more sleep interruptions in patients with poor PS compared to those with good PS (Table 6).

We evaluated several Cosinor parameters such as circadian amplitude, circadian fragmentation/amplitude of ultradian rhythms, circadian quotient, peak activity, activity/rest dichotomy (I<O), rhythm quotient to measure the relationship between circadian function and PS. The only circadian function that was statistically significant different in this small sample was the frequency of ultradian/4-h rhythms (*P*=0.046). Higher 4-h amplitudes indicate disruption of circadian rhythm, which normally has one peak.

The circadian quotient, which provides a measure of the strength of the circadian rhythm, compared to other components, suggests that circadian rhythms are more pronounced in patients with PS 0 or 1 than patients with PS 2. Daily activity of normal people usually has one or two circadian peaks, with best cosine fit at 24 or 12 or 12 and 24 h. The rhythm quotient provides a basis for assessment of the degree of activity/sleep consolidation within each day. Higher circadian rhythm quotient indicates a more pronounced circadian rhythm and lower values indicate a fractured circadian sleep/activity patterns. From Table 6, it is known that rhythm quotient has an inverse relationship to PS levels. This indicates that patients with scores of 0 or 1 have less fractured circadian sleep activity patterns. Furthermore, 24-h autocorrelations (*r*_24_) are higher in patients with PS 0, although not statistically significant, indicating more pronounced and reproducible day-to-day circadian activity rhythm.

**Figure 1:**
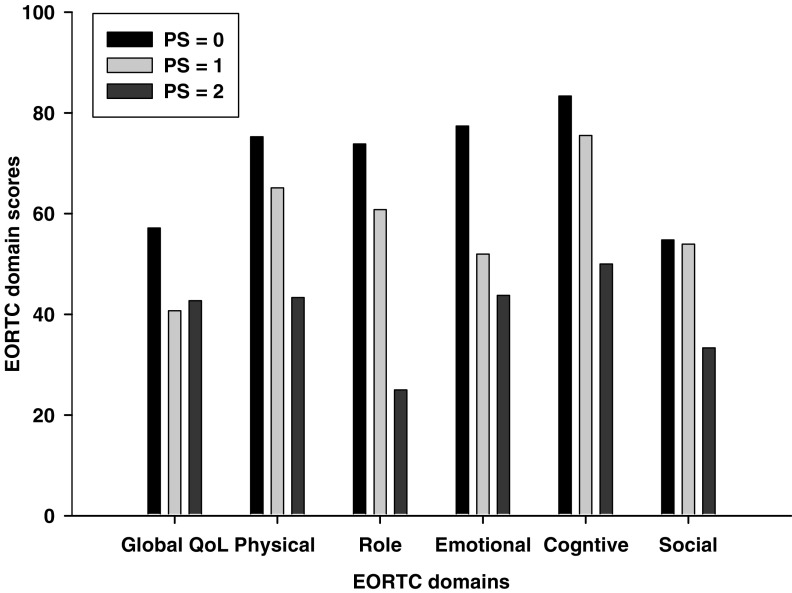
EORTC-QLQ-C30 domain scores by ECOG performance status.

There were systematic differences in the Quality of Life (EORTC-QLQ-C30) scores by PS. In all domains, patients with a PS of 0 scored over 20 points higher than the patients with PS score of 2 (Figure 1). Moreover, there were greater than 10 points differences in the physical, role, and emotional domains between PS 0 and 1 patients. Finally, there was a significant difference between the near normal fatigue score of 36.51±5.8 in PS 0, and the PS 1 and PS 2 scores of 54.9±7.8 and 70.83±9.6, respectively (Fc=3.70, *P*=0.037). All of these scores are high and, they increase with increasing PS scores.

**Figure 2:**
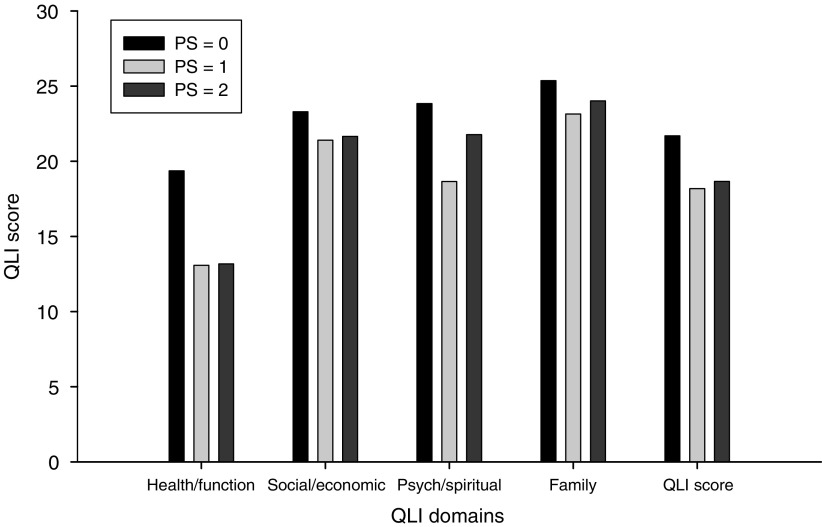
Quality of Life Index domain scores by ECOG performance status.

The second QoL instrument, the QLI, also detected differences by performance status scores. PS 0 patients had scores that were at least 1.9 points higher than patients with PS scores of 1 or 2 in all domains of the QLI instrument. Indeed, patients with PS score of 0 scored over six points higher in the Health/Functioning domain than patients with PS scores of 0 and 1 (Figure 2). Overall, PS 2 experience more intense symptoms, more difficulties in meeting the challenges of daily life, and express higher levels of dissatisfaction with Health Function.

## DISCUSSION

Non-small-lung cancer patients were recruited on a clinical trial that investigates whether exogenous melatonin at the appropriate circadian time improves health-related QoL, diminishes patient fatigue, and enhances sleep quality and quantity, by a statistically significant level. We report our analysis of the baseline status of the initial 33 trial participants to determine whether patients with advanced NSCLC have distorted circadian rhythms, poor sleep quality, and compromised QoL. We did this analysis because there is little data in the clinical literature on the circadian function of cancer patients, in general and NSCLC patients, in particular (Hrushesky *et al*, 1985; Mormont *et al*, 2000). Moreover, the literature has even fewer data on the relationships between circadian function, PS, and QoL.

Our analysis found that patients with advanced NSCLC have distorted circadian rhythms. Trial participants experienced higher than expected levels of wakefulness during the normal sleep period and surprisingly extensive sleep periods during normal times for activity. The patients, for example, spent 71.2% of the putative sleep time ‘sleeping’, while a ‘normal person’ spends 93% of the putative sleep time ‘sleeping’. Conversely, these good PS cancer patients were sleeping 21.8% during the putative wakefulness time, while normal people spend only 4.7% of the day completely inactive. All other actigraphy data items confirm this pattern of excess rest during the activity phase of the circadian cycle and increased activity during the rest phase of the daily cycle of sleep and activity. Cosinor analysis shows clearly that our patients' circadian rhythm function is significantly different from normal persons. However, because of the obvious age and gender differences between our patient cohorts and the reference population, we need age and gender-matched comparison to adequately evaluate the circadian patterns of our patient population.

Curiously, there was no correlation between a patients' PS score and any Actigraphy data item. PS is the most useful stratification variable for predicting patient response and survival (Karnofsky *et al*, 1948). For example, the potential lifespan of a PS 2 patient is considerably shorter than that of a patient with a PS of 1. The actigraphy data indicated that the occurrence of a stage III or IV, pretreated or newly diagnosed, produces severe significant impairment in the patient's circadian function. A circadian rhythm has a 24 h period. Cosinor analysis revealed that circadian rhythms of patients with PS 2 were more distorted than those of patients with a PS 0 or 1. Consequently, patients with more advanced disease experience a greater disruption in their circadian rest/activity cycle.

As expected, sleep quality data from the PSQI confirms the actigraphy data. Patients' PSQI Global Sleep Quality Score of 10.6 is well above the threshold level of five for poor sleep quality. With the exception of daytime dysfunction, the sleep instrument found no statistical differences by performance status scores. Consequently, both the actigraphy and PSQI reveal that patients with advanced NSCLC irrespective of their treatment history have very poor sleep quality. More importantly, the very poor sleep quality of these patients was independent of the patients' PS score. This means that, by the time advanced lung cancer patients present for therapy, their circadian organisation has been greatly disturbed.

Clinical research among noncancer patients has linked poor sleep quality with lower scores in instruments measuring health-related quality of life. The self-report of QoL among participants of this trial were significantly lower than the general population. The mean scores for the EORTC functional domains, with the exception of the cognitive domain, were over 20 points less than those reported in population-based surveys. Moreover, in the EORTC symptom items, our trial participants reported greater than 20-point increase (worse symptoms) in a variety of EORTC symptom items (pain, fatigue, dyspnoea, insomnia). These data make it clear that the trial participants' ability to fulfil the activities of daily life is much lower than that of the general population.

The EORTC instrument emphasises the patients' capacity to perform the activities of daily living, but the QLI evaluates the patients' degree of satisfaction with their QoL. The trial participants' Health and Function domain score is at the bottom quintile of the general population. Thus, our patients are expressing a high degree of dissatisfaction with their capacity to fulfil the activities of daily life. Interestingly, despite the difficulties the trial patients were experiencing in meeting the challenges of daily living, they still expressed satisfaction with key aspects of life – social and economic, psychological and spiritual, and family that is similar to the general population.

Unlike the actigraphy and PSQI data, the PS scores stratified the QoL results. The EORTC functional domains scores were highest in patients with a PS 0 status and lowest in PS 2. Moreover, the symptom items tended to increase with increasing PS scores and the fatigue scores for PS 2 patients were twice the scores reported by PS 0 patients. The QLI instrument separated patients with PS 0 but did not differentiate PS 1 and 2 patients.

Significant differences between the two study sites were observed in almost all of the circadian parameters. We attribute this to the different millieux when actigraphy is measured. Actigraphy was obtained in VAMC patients in the ambulatory/home setting prior to therapy and in hospital during chemotherapy in MRMC patients. As a result, the MRMC patients might have been more sedentary than usual. Circadian rhythms of patients in MRMC are less robust, day–night activity difference is less pronounced. Further, day-to-day rhythm of VAMC patients is more stable compared to MRMC, indicated by the 24-h autocorrelation.

In summary, patients with advanced NSCLC have distorted circadian function independent of their PS. Indeed, the development of an advanced NSCLC is accompanied by very unsubtle distortions in an individual's circadian function. Moreover, the patients exhibit the attributes of individuals suffering from disrupted circadian function – complaints of poor sleep quality, fatigue, and insomnia. We also documented the superiority of outpatient/home as opposed to inpatient actigraphy for most accurately measuring and assessing the circadian organisation of cancer patients.

Several limitations of this study require careful acknowledgment. This interim analysis suffers from relatively small sample size. Our sample size is not enough to accommodate the number of comparisons made within this study. There were clear demographic and clinical differences between the two populations recruited at the two participating cancer centres making our overall study population a little heterogeneous. Lastly, we lacked adequate age- and gender-matched comparisons to clearly evaluate the extent of distortions in circadian function of our cancer patients. Despite these limitations, we believe that the participants of this trial are appropriate subjects to investigate whether exogenous melatonin administered chronotherapeutically can restore patient circadian function, improve sleep quality, health-related quality of life, and survival.

## Figures and Tables

**Table 1:** Sleep and activity during putative daily wake span

**Sleep and activity variable**	**Cancer patients (*n*=33)**	**Reference population (*n*=35)**
Mean daily activity (min)	92.8±5.63	127±17.11
Mean activity during wakefulness (min)	117.5±7.11	182.6±25.08
Mean duration of sleep during wakefulness (min)	195.0±25.03	46.5±41.05
% of wakefulness spent sleeping	21.8±2.93	4.7±3.92
Number of sleep episodes during wakefulness	17.8±1.82	5.4±5.06

**Table 2:** Sleep and activity during putative daily sleep span

	**Mean and standard error**	
**Sleep and activity variable**	**Cancer patients (*n*=33)**	**Reference population (*n*=35)**
Frequency of long naps	9.5±1.08	2.1±1.96
Duration of wakefulness during night sleep (min)	134.1±14.94	31.1±21.53
Number of sleep interruptions nightly	14.6±1.35	6.9±4.52
% of sleep span actually spent sleeping	71.2±3.09	93.0±4.88
Nightly average duration of long sleeps (min)	112.5±13.22	225.6±100.8

**Table 3:** Pittsburgh Sleep Quality Index (PSQI) (*N*=33)

	**Study population**	**Healthy controls^a^**
**Sleep indicators**	**Mean±s.e.**	**Mean±s.e.**
Sleep quality	1.7±0.18	0.35±0.07
Sleep latency	1.6±0.22	0.56±0.10
Sleep duration	1.3±0.22	0.29±0.07
Sleep efficiency	1.3±0.24	0.1±0.04
Sleep disturbance	2.3±0.12	1.0±0.05
Sleep medication	0.9±0.19	0.04±0.04
Daytime dysfunction	1.5±0.27	0.35±0.07
Global sleep quality score	10.6±0.95	2.67±0.23

a Owen *et al* (1999) 26, 1649–1651.

**Table 4:** EORTC QLQ-C30 – quality of life (*N*=33)

**Domain**	**Study data mean**	**Population-based survey data mean**	**Difference in mean scores^a^**	**EORTC data on advanced non-small-cell lung cancer**
*Functioning scales*				
Physical function	64.2	89.9	−25.7	64.6
Role function	54.8	83.3	−28.5	67.2
Emotional function	50	82.8	−32.8	67.2
Social function	51	85.8	−34.8	70.1
Cognitive function	72.8	86.5	−13.7	82.7
Overall QLI	45.5	75.3	−29.8	57

*Symptom scales*				
Fatigue scale	53	28.8	24.2	42.5
Nausea	13.8	4	9.8	12.5
Pain scale	59	20.5	38.5	30.5

*Single items*				
Dyspnoea	45.7	14.3	31.4	41
Insomnia	42.8	20.4	22.4	33
Appetite loss	36.2	7.5	28.7	35.4
Constipation	32.4	10.4	22	23.3
Diarrhoea	9.5	9.4	0.1	4.7
Financial difficulties	40.2	9	31.2	13.6

a Difference between Study patients and population-based survey data.

**Table 5:** Ferrans and Powers QLI (*N*=33)

**Domain**	**Study population – mean scores**	**Population-based mean scores (std)**	**Difference in mean scores**
Health/function	15.36	23.19 (4.47)	−7.83
Social and economic	22.06	21.83 (4.11)	0.23
Psychological/spiritual	21.4	22.95 (5.21)	−1.55
Family	24.08	25.60 (4.49)	−1.52
Overall quality of life score	19.65	23.00 (4.04)	−3.35

**Table 6:** Actigraphy data

**Variable**	**Performance status 0**	**Performance status 1**	**Performance status 2**
Mean activity during wakefulness	123.92±19.84	114.38±9.13	118.61±13.21
Number of sleep interruptions	7.49±1.34	9.09±1.31	11.5±2.82
Nightly average duration of long sleep	128.99±26.15	112.24±20.36	96.52±20.36

*Rhythm parameters*			
24-h amplitude	54.17±10.05	47.58±3.45	42.99±4.40
Amplitude of 4-h cosine fit	8.11±1.46	7.86±1.05	12.86±1.86
Circadian quotient	0.55±0.08	0.53±0.04	0.47±0.06
Rhythm quotient	1.05±0.16	1.02±0.09	0.94±0.16
24-h autocorrelation	0.31±0.06	0.17±0.04	0.21±0.05

## Appendix I

The following parameters described the activity phase of the daily circadian cycle: mean daily activity, mean duration of activity during wakefulness, mean duration of sleep during wakefulness, proportion of wakefulness spent sleeping, number of sleep episodes during wakefulness, and frequency of long naps. During the presumed sleep phase of the circadian cycle, the following parameters were evaluated: mean duration of wakefulness, number of sleep interruptions, proportion of sleep episode spent actually sleeping, and frequency of long duration of sleep.

The number of accelerations per minute for a continuous 4–7 days was recorded through the actigraphy watch and was translated into sleep/activity parameters through the Act Millennium and Action W2 software (Ambulatory Monitoring, Inc.). Rhythmometric analysis (using Chronolab v2) was carried out on these sleep/activity patterns in order to assess disruption and consolidation of sleep in lung cancer patients. Rhythmometric analysis fits a cosine curve to the circadian activity providing three standard parameters: mesor – the average activity over the 24-h period, amplitude-peak to nadir difference and acrophase – the time of peak activity. In addition to these parameters, peak activity (mesor+amplitude) to measure activity levels, the circadian quotient (amplitude/mesor) to characterise the strength of the circadian rhythm, and the rhythm quotient (*A*_24 h_/(*A*_4_+*A*_8_+*A*_12_)) were computed. Higher amplitudes indicate more robust rhythms but people who move vigorously would have higher amplitude, thus, the circadian quotient provides normalised values that would allow comparison between individuals (Satlin *et al*, 1991; Ancoli-Israel *et al*, 1997). We also looked at the dichotomy index (I<O), comparing amounts of activity when in bed and out of bed, which is significantly associated with colorectal cancer patients' quality of life and survival (Mormont *et al*, 2000). I<O is the percentage of minutes or epochs during the putative sleep span with activity score that are less than the median activity during the putative wakefulness. Thus, high I<O reflects a marked rest/activity rhythm (Mormont *et al*, 2000). Further, circadian rhythms were assessed through spectral density analysis where 24-h autocorrelations (*r*_24_) were computed. Autocorrelations theoretically can range from −1 to +1. If a circadian variation is present, autocorrelations will increase around 24 h and a more pronounced and stable day-to-day circadian rhythm will result in a higher autocorrelation at 24 h.
